# Bacterial biopesticides: Biodiversity, role in pest management and beneficial impact on agricultural and environmental sustainability

**DOI:** 10.1016/j.heliyon.2024.e31550

**Published:** 2024-05-18

**Authors:** Preety Tomar, Neelam Thakur, Samiksha Jhamta, Sohini Chowdhury, Monit Kapoor, Sangram Singh, Sheikh Shreaz, Sarvesh Rustagi, Pankaj Kumar Rai, Ashutosh Kumar Rai, Ajar Nath Yadav

**Affiliations:** a Department of Zoology, Akal College of Basic Sciences, Eternal University, Sirmour, Himachal Pradesh, India; b Chitkara Center for Research and Development, Chitkara University, Himachal Pradesh, India; c Centre of Research Impact and Outcome, Chitkara University, Rajpura, 140401, Punjab, India; d Department of Biochemistry, Dr. Ram Manohar Lohia Avadh University Faizabad, Uttar Pradesh, India; e Desert Agriculture and Ecosystems Program, Environment and Life Sciences Research Center, Kuwait Institute for Scientific Research, PO Box 24885, 13109, Safat, Kuwait; f Department of Food Technology, School of Applied and Life Sciences, Uttaranchal University, Dehradun, Uttarakhand, India; g Department of Biotechnology, Invertis University, Bareilly, Uttar Pradesh, India; h Department of Biochemistry, College of Medicine, Imam Abdulrahman Bin Faisal University, Dammam, Kingdom of Saudi Arabia; i Department of Genetics, Plant Breeding and Biotechnology, Dr. Khem Singh Gill Akal College of Agriculture, Eternal University, Baru Sahib, Sirmour, Himachal Pradesh, India

**Keywords:** Agricultural sustainability, Biopesticides, Crop protection, Pathogen, Pest

## Abstract

Agro-environmental sustainability is based upon the adoption of efficient resources in agro-practices that have a nominal impact on the ecosystem. Insect pests are responsible for causing severe impacts on crop productivity. Wide ranges of agro-chemicals have been employed over the last 50 years to overcome crop yield losses due to insect pests. But better knowledge about the hazards due to chemical pesticides and other pest resistance and resurgence issues necessitates an alternative for pest control. The applications of biological pesticides offer a best alternate that is safe, cost-effective, easy to adoption and successful against various insect pests and pathogens. Like other organisms, insects can get a wide range of diseases from various microbes, such as bacteria, fungi, viruses, protozoa, and nematodes. In order to create agricultural pest management practices that are environmentally beneficial, bacterial entomopathogens are being thoroughly studied. Utilization of bacterial biopesticides has been adopted for the protection of agricultural products. The different types of toxin complexes released by various microorganisms and their mechanisms of action are recapitulated. The present review described the diversity and biocontrol prospective of certain bacteria and summarised the potential of bacterial biopesticides for the management of agricultural pests, insects, and other phytopathogenic microorganisms in agricultural practices.

## 1 Introduction

Transforming the recent agriculture system with the aim of providing better food resources to the ever growing population is quite challenging. According to the latest estimation from the United Nations (UN), by 2050, the world population might reach up to 9.7 billion [1]. Although the global food supply is quite enough to meet the food requirements for the current population, it must be increased to fulfil the feeding requirements of the upcoming generations for the next decade [2]. In developing nations, due to increased earnings, food habits such as proteins, meat, and also demand increased food production. By 2050, food requirements will also rise from 59 % to 98 %. So there is a need to raise crop productivity worldwide [3].

About 20–40 % losses in yield have been encountered through diseases and insect pests annually [4]. Over 30,000 weeds, 10,000 insect species, 1,000 nematode species and more than 1,00,000 diseases caused by microorganisms have a major impact on agriculture and lead to losses in yield annually [5]. Even in India, insects, pests, weeds, and diseases caused by them are responsible for huge crop losses in yield every year [6]. Efforts have been made to overcome or eradicate the pest population by applying chemical compounds (insecticides, pesticides, nematicides, and weedicides [7]. The use of these chemical pesticides has been a common technique to manage the pest population over the past century. Although this technique is quite successful in managing the pest population, excessive application of these chemical pesticides leads to soil degradation, environmental hazards, pesticide residues in food, resistance among pests, and human health issues [8].

Excessive use of synthetic pesticides accounts for around 39 % of food crop losses throughout the world [9,10]. Even non-targeted insect pests have also been deadly affected by these pesticides and have had a hostile effect on the food chain [[11], [12], [13]]. So an alternate method is required to overcome the problems caused by synthetic chemical pesticides. The development of biopesticides is an alternative way to get rid of these harmful chemicals [14]. Biopesticides comprise a series of naturally occurring living substances and their metabolic products that are used to control the population of pests by adopting different strategies [15]. According to the Environmental Protection Agency (EPA) of the United States, biopesticides are insect pathogenic microbiomes that were obtained from natural resources and possess insect killing ability through infection [16].

In crop protection sciences, a biological pesticide plays an imperative role in the world. The idea of using alive organisms and their by-products against many pests provides support to the natural environment as it is safe and sustainable alternative to chemicals [17,18]. Biopesticides obtained from the microbiome (bacteria, fungi, nematodes, viruses and protozoan) can be widely used to manage the population of insects, nematodes, weeds and plant pathogens [[19], [20], [21], [22], [23], [24], [25]]. About 74 % of biopesticides worldwide are obtained from bacterial microbiomes [26]. Insect pathogenic bacterial species, commonly known as entomopathogenic bacteria (EPB), are single celled creatures that have the potential to infect and kill arthropods such as insect pests, and mites [27,28]. Numerous bacterial species of EPB have the ability to cause infection as well as damage to insects [29]. Applications of EPB have been practiced under the Integrated Pest Management (IPM) program which is widely employed for the control of insect pests [30]. Entomopathogenic bacteria are of two different kinds, i.e., endospore producing and non-endospore producing bacteria.

Actinobacteria is the principal phyla that possess bacterial as well as fungal natures [31]. In sustainable agricultural practices, these Actinobacteria could be act as biological control agents instead of chemical pesticides [32]. Many insect-killing compounds, namely polynactins, avermectin, spinosyns, abamectin, milbemycin and emamectin were isolated from Actinomycetes and have the potential to cope with insect pest problems in agriculture [33]. Several strains of Actinobacteria have been used as potential biocontrol agents (BCAs) against *Rhizoctonia solani* and *Macrophomina phaseolina* [34]. A compound macrotetrolides that has been isolated from *Streptomyces* species is found to be very effective against numerous insects, mites and termites [35]. Under the family Bacillaceae, the most important and well-known entomopathogen is *Bacillus thuringiensis*. In the last few decades, crystalliferous strains of *Bt* are highly recommended for the control of pest populations [36]. Due to cry proteins Bt has been used for the management of insect pests belonging to the order Diptera, Lepidoptera and Coleoptera [37,38]. Lactonase released from Bt reduces the pathogenic effect caused by plant pathogenic bacteria. *Bacillus thuringiensis* also produces zwittermicin which has antifungal as well as antibiotic properties [39].

Bacterial biopesticides are economical and extensively employed for the management of pest populations. Various types of insect pests were affected by numerous bacterial species [40]. Biopesticides are actually selective and safe for the environment as well as for non-targeted living organisms [41]. These have the capacity to manage the pest population and also show different approaches to cope with pest resistance [9]. Biopesticides are economical and cheap, easy to apply, long lasting, very effective, eco-friendly, and show a variety of action mechanisms.

The highest proportion of biopesticides is consumed in North America (44 %) followed by Oceania and the EU (20 %) and South America (10 %), while Asian countries such as India account for only 6 % consumption [7]. Biopesticides derived from microorganisms, mainly EPB ruled over the entire biopesticides industry worldwide. The bacterial biopesticides are easy to produce, highly specific, long lasting, reside in the environment permanently, and are harmless. Since the 20th century or earlier, bacterial biopesticides have been used as biocontrol agents but only a few bacterial biopesticides have been commercialized to control the population of pests to date. This review included the diversity and biocontrol prospective of certain bacteria and summarised the potential toxins from bacteria responsible for the management of agricultural pests, insects, and other phytopathogenic microorganisms in agricultural practices.

## 2 Biodiversity of entomopathogenic bacteria

The first effort to apply bacteria for insect management was made by Felix d’Herelle [42]. A single EPB, *Bacillus thuringiensis* (Bt), produces about 90 percent of total bacterial biopesticides [43] other groups of entomopathogenic bacteria include *Pseudomonas*, *Chromobacterium*, and *Yersinia* [44], also contributes in the production of bacterial biopesticides. Insecticidal toxins have been produced by EPB that aggregate into cellular inclusion, leading to insect death, as well as secrete toxic substances that have insect killing potential [45]. Bt as a bio-insecticide was used by the French in early 1920, while the first commercial formulation of Bt (Sporeine) became available on the market in 1938. In the United States, the use of biopesticides appeared in 1950s. High scale production of *Bt* was done by Pacific Yeast Product Company through the submerged fermentation process [46]. In India, under the Insecticide Act 1968, merely 12 kinds of biological pesticides have been registered [47]. Among these, only Bt is considered the most essential biological control agent for pest management throughout the world [48]. During the investigation of sudden collapse disease in Japan, EPB was first detected in the infected larvae of *Bombyx mori* [49,50]. This investigation on EPB laid the basis for biopesticide exploration of other insect killing bacteria. *Pseudomonas fluorescens* (Trevisan), *Bacillus subtilis* (Ehrenberg), and *Pseudomonas aureofaciens* (Kluyver) are also used against a variety of plant pathogens in agricultural practices [51].

Crystalliferous spore formers, obligate, facultative, and potential pathogens are four different kinds of bacteria that are used to produce various biopesticides. Biopesticide producing bacteria such as crystalliferous spore formers include *B. thuringiensis*, *B. thuringiensis* subsp. *kurstaki, B. thuringiensis* subsp*.* tenebrionis, *B. thuringiensis* subsp*. aizawai* and *B. thuringiensis* subsp*. israelensis* that are effective against Lepidopteran insect larvae (armyworms and Diamondback moth) mosquito's larvae, simulids and also against several coleopteran insects. *Lysinibacillus sphaericus* is used to control the population of mosquitoes (Culex and Anopheles) through the production of binary (Bin) toxin [52]. *Saccharopolyspora spinosa* is used against a variety of insect pests including *Aedes aegypti* and *Spodoptera littoralis* [53,54]. *Pseudomonas aeruginosa* are facultative pathogens while *Bacillus popilliae* are the obligate pathogens responsible for the production of biopesticides [55]. Among all these four categories of bacteria, crystalline spore forming bacteria are predominantly used in the commercial formulation. Due to their effectiveness, efficiency and safety purposes, *B. thuringiensis* and *B. sphaericus* are widely adopted for insect pest management [56]. The EPB also resists symbiotic mutualism with entomopathogenic nematodes (EPNs) and shows insecticidal properties. These bacteria belong to the genera *Photorhabdus* and *Xenorhabdus* [57].

Bacteria *Brevibacillus laterosporusm* is a Gram positive organism first recognised in 1912 that produces a parasporal body canoe-shaped. *Chromobacterium substugae* as a biocontrol agent was tested against the larvae of *Leptinotarsa decemlineata* (Colorado potato beetle) in Maryland, USA [58]. *Yersinia entomophaga* was first accessible from New Zealand in 2011. This species has the potential to manage a broad range of agricultural insect pests, including insect orders such as Lepidoptera, Coleopteran and Orthoptera [59]. Carl Woese in 1980 first defined the phylum Proteobacteria as Gram negative bacterial phylum that is further classified into alpha, beta, gamma, delta, and epsilon. All these classes possess bacterial species that have the potential to suppress and kill insect pests.

## 3 Mechanism of action

**Fig. 1:**
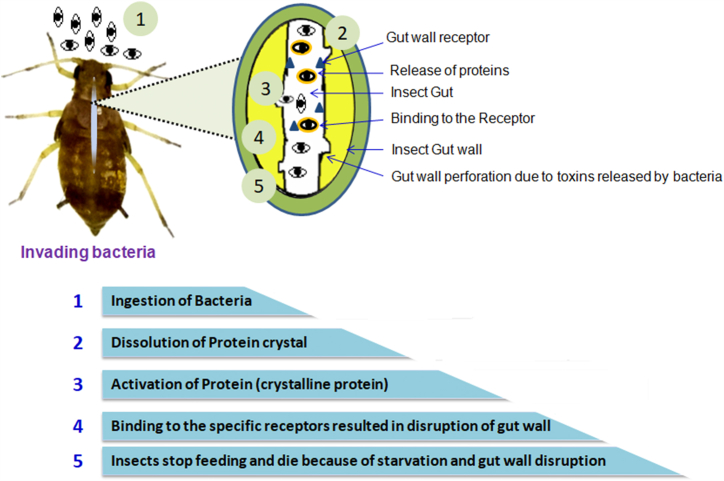
Mode of action by bacterial biopesticides.

The most important and well-known entomopathogen in the world is *B. thuringiensis*. In addition to creating crystalline proteins, *B. thuringiensis* eradicates wide range of target insect pest species, including lepidopteran species [60]. Bt exhibits some degree of toxicity during sporulation through the creation of endotoxins. Crystalliferous inclusions react with receptors on epithelial cells in the midgut to release δ-endotoxins, which kills insect pests [61]. Nevertheless, the endotoxins have to be eaten by the larvae in order for the desired level of toxicity to be reached; this leads to the destruction of the gut tissues, which in turn induces gut paralysis [62]. *Photorhabdus* and *Xenorhabdus* bacteria, the mode of entrance into the host is very different. The two bacteria coexist in a mutualistic relationship with dauer juvenile entomopathogenic nematodes. Nematodes enter the host body through spontaneous holes, releasing bacteria either through oral pathways or through faeces inside the insect's hemocoel [63]. The bacterial cells proliferate and create potent toxins that have a great potential for insecticidal actions [64]. The bacterially generated proteinaceous toxins during the late infectious stage of the insect damage its midgut [65,66] (Fig. 1).

### 3.1 Compounds produced by bacteria

Many bacterial species produce a huge amount of secondary metabolites that coexist in dynamic communities, which help them to accept biotic and abiotic stress [67]. A diverse population of bacteria such as *Pseudomonas*, *Bacillus* and *Streptomyces* releases more than 1,000 volatile bacterial compounds [68]. These compounds show several antimicrobial activities and have different antimicrobial properties with Gram-positive bacteria [69] and Gram-negative bacteria, [70]. These bacteria produce and release a wide range of diverse organic and inorganic volatile compounds, such as nitrogen containing compound, sulfer, terpenes, hydrocarbons and some inorganic compounds like nitric oxide (NO), hydrogen sulfide (H_2_S), ammonia, or hydrogen cyanide (HCN) [71]. The compounds produced by *Photorhabdus* and *Xenorhabdus* bacteria include both volatile and soluble compounds and have biological and physiological properties. These compounds usually show stronger biological activities as antibiotics or toxins as a result of their maximum degree of functionalization [72]. On the other hand, volatile organic compounds are small molecules [73] and soluble compounds have a higher polarity, which makes them soluble in water. Therefore, these EPB are commonly characterised as unique and rich sources of secondary metabolites that develop toxins (proteins) with low molecular weight that are used against insect pests or some other eukaryotes and prokaryotes.

### 3.2 Toxins produced by bacteria

The toxins released by bacteria are the most influential poisons in nature [74]. These toxins are able to perform several important tasks, like modification of cellular components by utilizing and learning cellular processes. *Chromobacterium subtsugae*, *B. thuringiensis,* and *B. laterosporus* are some examples of bacteria where toxins are needed for insect death. Some important proteins produced by these EPB are the Cry, Cyt, Vip and Bin toxins [75]. Amongst these toxins, Cry toxins are the most characterized and studied (Table 1).

**Table 1:** Entomopathogenic bacteria and their toxin complexes.

Bacterial Species	Description	Toxin Produced	References
***B. thuringiensis***	Crystal toxins produced during sporulation, pore forming toxins that act on gut columnar cells	Cry toxins	[163]
	Pore forming toxin that acts on gut columnar cells and causes death to insect brains cells	Cry1C toxin	[164]
	Associated with the presence of certain cry and vip genes, toxic to mammalian cells, growth inhibition and secreted during vegetative growth cycle	Ǻ-exotoxin I	[165]
	Vegetative insecticidal protein produced during the vegetative growth cycle, binds to midgut receptors causes gut paralysis and lysis in midgut columnar cells	Vip3 toxin	[166]
***B. subtilis***	Biosurfactant, destructive effect on gut epithelial cells by vesicle formation in the apical region and cellular vacuolization	SPB1	[167]
***L. sphaeriscus***	Cholesterol binding cytolysin, lyses neurons and hemocytes	Sphaericolysin	[168]
***B. cereus***	Secreted non-proteinous exotoxins, pathogenic to humans and insects	Exotoxin	[169]
***B. thuringiensis israelensis***	TcaCBA and TccC toxin homologs to *Photorhabdus* and *Yersinia*	TcaCBA and TccC toxin	[170]
***Clostridium difficile***	Exotoxin which reorganizes cytoskeleton, hydrophobic central domain is shared between the Mcf1 and Mcf2 toxins and are cytotoxic motif	Cytotoxin B	[171]
***Clostridium botulinum***	Binary actin binding-ADP-ribosylating exotoxin	C2 toxin	[172]
***S. entomophila***	Shows high similarity to *P. luminescens* TcbA, TcdA, TcaB, and TccB, tripeptide cell-binding motif Arg-Gly-Asp implicates that shows the ability to bind with eukaryotic cells	SepA	[173]
	Shows similarity with *P. luminescens* TcaC and SpvB	SepB	[173]
	Shows similarity to *P. luminescens* TccC, also Shows similarity with *B. subtilis* WapA, *E. coli* Rhs, *Streptomyces coelicolor* A3, *Coxiella burnetii*	SepC,	[173]
***Yersinia frederiksenii***	Antifeeding prophage causes cessation of feeding	Afp	[174]
	Orally toxic, not subcutaneously toxic and high homology to sep genes in *Serratia*	TcYF1 and TcTF2, TcA	[174]
	Assists TcC to translocate into the cell, transmembrane pore formation, contains an integrin binding motif	TcdB1, TccC1, TcaC	[174]
***P. luminescens***	Potentiates expressed toxin genes tcdA1, tcaA and tcaB	TcdB2, TccC3	[175]
	Assists TcC (TccC3) to translocate in the cell	TcB (TcdB2)	[176]
	Actin-clustering, defects in phagocytosis and cell death	TcC (TccC3)	[177]
	Formation of actin aggregates and ADP-ribosylates actin at the threonine148 with TccC3 sub-complex	Tc Complex	[178]
	Makes caterpillars floppy phenotype, induce apoptosis and rearrange actin	Mcf	[179]
	Degrades peritrophic matrix, causes midgut damage, midgut cell sloughing and causes fat body nuclear degradation	Txp40	[180]
	*Photorhabdus* virulence cassettes, phage-like, kills and condenses actin of hemocytes	PVC	[181]
	Primarily hemoplymph-based insecticidal activity	pirA2B2, locus, plu4437-plu4436	[182]
	Similar to *Serratia*-type hemolysins	phlBA operon	[183]
	Binds actin ADP-ribosylating and inhibits actin polymerization	Photox	[184]
	Midgut and intestinal sloughing	A24tox	[185]
***X. nematophila***	Alphaxenorhabdolysin triggers apoptosis in hemocyte cells, cytotoxic and haemolysin effects, has analogs in *Photorhabdus, P. entomophila, P. syringae, Y. enterocolitica* and *Proteus mirabilis*	xaxAB	[186]
	From the cosmid CHRIM1, analogs to sep *Serratia* genes and causes oral toxicity	XptA1, XptA2, XptB1,XptC1	[187]
	Fed to neonates and caused inhibitory growth	Xin	[188]
	High similarity to GroEL, injectable toxicity to *G. Mellonella* and innate	xaxAB,	[186]
	Immune response of increased phenoloxidase activity stimulated by injection	HIP57	[189]
	One gene in the xenocin operon with RNAse and cytotoxic activity	xciA	[190]
***P. fluorescens***	Related to the Mcf from *P. luminescens* that shows hemolymph-based insecticidal activity Regulators of Fit insect toxin expression for biocontrol	Fit	[191]
***P. entomophila***	Exotoxins with hemolytic activity Lipases	TcdB and Tcc-C type toxins	[170]
***P. syringae***	Oral toxicity	TcdB and Tcc-C type toxins	[192]

#### 3.2.1 Cry toxins

The Cry proteins are produced as secretary proteins by *B. thuringiensis* but have also been recognised by the other bacteria [76] that are also encoded with cryptic genes [77]. The amino acid sequence is currently used as the basis for the nomenclature and classification of Cry toxins such as Cry1, Cry2, and Cry3 [78]. Cry toxins like Cry51Aa, Cry15, Cry23, Cry33, Cry45, and Cry46 present a single beta-stranded domain and show resemblance to aerolysin-type beta-pore forming toxins. These Cry protein toxins showed unique toxicity against hemipteran and coleopteran pests [79]. Although these structural similarities suggest commonalities with aerolysin-type toxins, the mode of action of these Cry toxins has not been experimentally characterized.

#### 3.2.2 Vip proteins

Vip proteins are produced by the vegetative cells of the host during the later stages of infection. Many workers suggested that these proteins are active after ingestion, and the main target of Vip proteins may be located in the heamocoel. When evaluated with the three domains of toxin, the low structural sequence of the Vip protein toxins sustains differences in the intoxication process. Vip1 and Vip2 function as A/B binary toxins [80], whereas Vip3 toxins are possibly responsible for pore formation because of their cytotoxicity [81]. In contrast to Cry proteins, Vip3 toxins do not have a protease-resistant toxin core. Therefore, ribosomal protein has been reported as a functional Vip3A binding protein and receptor in Sf21 insect cell cultures [82].

#### 3.2.3 Bin toxins

*Lysinibacillus sphaericus* produces Bin toxin, which is released as a single parasporal crystal containing the same molar concentration of two proteins of 42 kDa (BinA or P42) and 51 kDa (BinB or P51) [83]. BinA and BinB genes are particularly preserved among the *L. sphaericus* strains, which have a ∼35-kb operon that is positioned equally on the chromosome and on the pBsph plasmid [84]. These two toxins have less sequence similarity but contribute numerous regions for identity that are essential for toxicity [85]. Bin toxins produced by *L. sphaericus* strains used in field applications are Bin 1 to Bin 4 [86].

#### 3.2.4 Toxin complex

Some EPB produce insecticidal toxic proteins, in general, Tc toxins are composed of three subunit proteins such as TcA, TcB and TcC. These proteins merge with each other to form the insect-active complex [87]. The TcC subunits have ADP-ribosyl transferase activity, which is responsible for cytotoxicity, and are situated inside a large hollow structure formed by the TcB and TcC subunits [88]. Once the binding of the complex is endocytosed, the TcA pentamer forms a slightly conical canal with an activated pH [89]. In which the cytotoxic domain of TcC is translocated in the cytoplasm through a syringe-like mechanism of protein translocation [90].

#### 3.2.5 Mtx toxins

Some strains of *L. sphaericus* release mix toxin, a mosquitocidal soluble protein in the vegetative phase [91]. These mix toxin proteins have a C-terminal 70-kDa fragment with sequence similarity to the lectin-like binding component of ricin [92] and a N-terminal 27-kDa fragment with ADP-ribosyltransferase activity [93]. This toxin is internalised into endosomes, wherever it experiences low pH, which can help in the translocation of the 27-kDa fragment into the cytosol to ADP-ribosylate proteins, which is hypothesised after the required interaction between the 70-kDa fragments with unknown receptors [94]. A mixture of Mix and Cry toxin from the subspecies of Bt causes a combined effect against *Culex quinquefasciatus* [95].

## 4 Commercial aspects and applications

The first commercial product in the market, Bt, was launched in 1938 in France under the name Sporeine. Till today, there has been a continuous increase in the development of Bt products. It was introduced as well as commercialized in the Thai market in 1965 [96]. In South Korea, pest control through microbial biopesticides was started in 1970, which involved the use of entomopathogenic bacteria, fungi, nematodes, and viruses to manage the population of pests in agriculture [97]. The first commercialized bio-pesticide, “Solbichae” was produced from Bt subsp. *aizawai*. This first bio-pesticide was registered in 2003 to manage the population of diamondback moths and armyworms in cabbage [98]. In Australia, microbial control began in the late 1960s with the GV of *Cydia pomonella,* codling moth and nucleopolyhedrosis virus (NPV) of *Helicoverpa zea*. Whereas, in Argentina, products of Bt were used for the first time in 1950 to control *Colias lesbia* in Alfalfa [99]. The commercial Bt products usually consist of crystal proteins, enzymes, viable spores, which are insecticidal in nature, along with other unknown virulent factors and adjuvants [100]. In India, the concept of biocontrol has been in practice ever since, while neem *Azadirachta indica* was used as a substitute for synthetic chemical pesticides. Farmers are using neem products not only for vegetable protection but also for numerous newly introduced medical applications [101]. In India, microbiopesticides grew as a nascent need when other insecticides were futile to manage the population of *S. litura*, *H. armigera,* and other insect pests of cotton [102]. In our country, commercial production of the biocontrol agents was started in Bio-Control Research Laboratories (BCRL), a division of Pest Control Limited, in collaboration with the Plant Protection Research Institute (PPRI) [103]. The intensification of biopesticides in India comes into existence when encouraged by the government as part of the IPM programme [104]. Commercial formulations of bacterial biopesticides are the most common form of microbial pesticides, which have prolonged storage potential, high residual activity, are economical, effective, and easy to apply against various insect pests.

Compatibility with cultural, chemical control approaches, and field application systems are the main necessities for the success of biopesticides [105], as is the need for the agricultural industry to adopt and accept the new technology. The composition and characteristics of developed formulations differ with the type of pathogen (type; regeneration mechanism; factors and characteristics), habitat (soil; water; size; foliage; warehouse), mode of application (land; aerial), rate of application (kg/ha and L/ha), insect species (life cycle; feeding niche; feeding habits), host-pathogen environmental connections (stability; resistance; behavioural changes), rheology of the technical material (particle size; density; viscosity), and mode of action (contact/oral). There are certain problems addressed by these bacterial formulations, such as loss of field activity and killing speed via the persistence of harmful environmental conditions such as adverse moisture (wet or dry), wind, rains, sunlight, plant characteristics such as the microbial development of opposing organisms, and modification of the application techniques by using inerts and adjuvants through chemistry [106].

### 4.1 Production of bacterial biopesticides

**Fig. 2:**
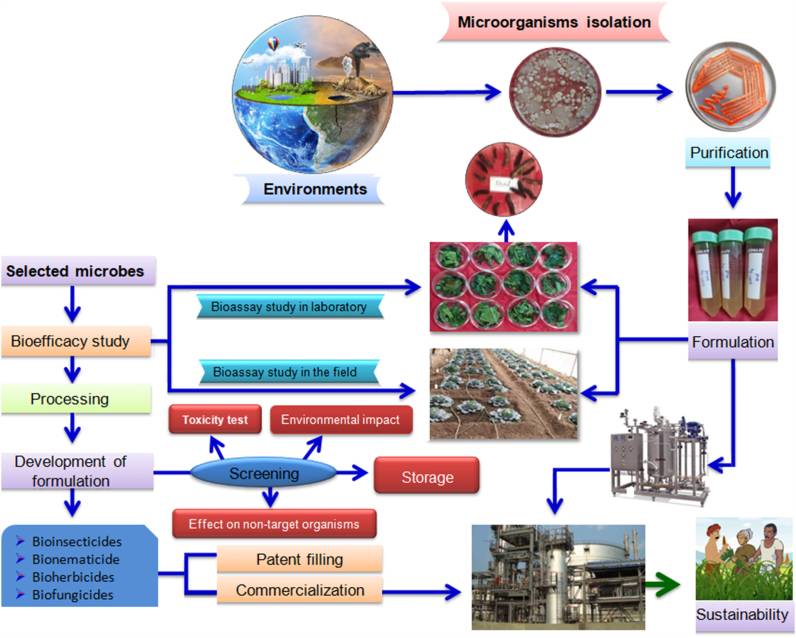
Production, application and commercialization of bacterial biopesticides.

There are numerous methods associated with the production and packaging of biological control agents. These methods include the production of dry as well as wet formulations [107]. Dry formulations include microgranular or macrogranular form, dust particles, powder form for seed coating, water dispersible granules, and wettable powder form (Fig. 2). These dry formulations have dilution properties that can be easily disseminated in water. Liquid formulations include ultra-low volume formulations, emulsions, capsule suspensions (CS), suspension concentrates (SC), suspo-emulsions (SE), and oil dispersions [108]. Generally, biopesticides are accessible in liquid, granular, and wettable powders on the market throughout the world [109].

The chemical processes such as absorption and adsorption were performed together by active ingredients (organisms) along with talcum powder, clay, to prepare “dusts” with very small particle sizes 50–100 μm. These dust powders used by hand or through others mean against the target organism. Several types of surfactants were also applied along with this dust powder to increase sorption [110]. Powders for seed coating” were invented to make an easy alliance between the formulation and seed covering by dropping the seed into the formulation [111]. Granular formulations were developed by the sorption of active ingredients along with other mineralizing resources such as attapulgite, ground plant residues, kaolin, dry fertilizers, silica, polymers, and starch in crude particle form, having a particle size of 100–600 μm for microgranular formulation and 100–1000 μm for macrogranular formulation [112]. These granular biopesticides release their active components after application and were used to control weeds, insects, and nematodes [113]. Several soil borne pathogens were managed by the application of vermiculite-based and lignite-based formulations [114]. Granules may be made with the help of extrusion [115], where the microbe is mixed with the clay or another carrier to make a paste and extruded to form in discrete pellets under pressure. In soil applications, the bacteria in the granule core can also be coated with seed [116].

To repress the problems raised by powder formulations, “water dispersible granules” were developed. These granules were spread in water and are dust-free with better strength. Extrusion granulation, spray drying, and fluid bed granulation are various techniques applied for the manufacturing of waterdispersible granules. These granules are much costlier but also much safer to handle [117]. Wettable powders were prepared by mixing and grinding the organism with a wetting agent, surfactant, or inert filler. These comprise the active component that will be a dispersant, a free flow agent, a wetter, the dried TGAI, and filler [118]. These powders were causing many health issues, such as eye and skin irritation, so they were not much considered in pesticide formulations [108]. Emulsions containing liquid droplets were easily mixed with water suspensions before application [100]. Several studies have been carried out to monitor a range of emulsifiers in order to recover preliminary invert emulsion formulations with BCAs [119]. Suspo-emulsions are demanding formulations formed by the fusion of the emulsion with the suspension concentrate, which decreases the difficulty of heteroflocculation between oil droplets and solid material [117]. Capsule suspensions (CS), along with active components, were involved in capsule formation from cellulose, starch, gelatin, and other polymers [100,120]. Formulation of the microbes is possible by using encapsulation to cover the organism with a shielding material. Formation of the microcapsules is prepared by using multiorifice centrifugation, interfacial reactions, phase separation, and electrostatic encapsulation [107]. Encapsulation has been achieved by genetic engineering for producing *Bt* toxins in the alternate bacterium, which can consequently be dried for cell protection. After the microcapsules are produced, they should be formulated in a liquid or dry formulation [121]. Ultra-low-volume liquids contain high concentrations of active ingredients with high solubility, crop-compatible liquor encloses surface active components and control additives that are easy to use [111].

### 4.2 Application approaches of bacterial biopesticides

Biocontrol formulations are distributed by numerous means, depending on the mode of infection and survival nature of the pathogen. All these different methods of application comprise foliar application, soil application, seed treatment, and the workable amalgamation of various methods (Fig. 2).

#### 4.2.1 Seed treatment

Seed treatment was done by applying the BCAs before sowing. It may be pre-coated with formulations such as oil and polymer-based liquids or dry powders or combined with soil [122]. Several additives, such as gum (xantham and arable), were also added to elevate the sticking of biopesticides to the seed coat. Seed encapsulation is also a method in which seeds coated with an active ingredient are covered by a polymer gelmatrix or gelatinous substances to enhance and extend the existence of microbial agents on the seed. An example of such seed encapsulation is GEL-COATTM alginate hydrogel capsules developed from entomopathogenic nematodes as a biopesticides. The application of these seed encapsulations is advantageous as they are eco-friendly and safe [123]. Even other formulations, such as fine dust powders, liquids, or wettable powders, may also be used at the time of plantation, with or without the sticky materials. This method is the most effective way of applying biocontrol agents, chiefly to counter soil-borne pathogens. The hydration of the seed is measured through techniques like seed preparation, which allows pre-germinative metabolic actions and avoids the appearance of radicals. Treatment of chickpea seeds and pigeon peas with a talc-based formulation (*Bacillus subtili*s) supports the control of *Fusarium* wilt in many crops [124,125].

#### 4.2.2 Soil treatment

Biocontrol agents can be applied to the soil prior to plantation or sowing, called post-fumigation treatment, which is quite effective [126] and results in a reduced soil pathogen population. This type of soil treatment with BCAs is called “suppressive soil,” in which only beneficial microorganisms stay in living condition [127]. Powder formulations, dust, and other granular products can be sprinkled directly into the soil while several liquid formulations were applied in channels [128]. The soil application by formulation of peat-based along with *P. fluorescens* (Pf1) at a dose 2.5 kg of formulation with 25 kg of farmyard manure (well-decomposed) enhanced management of the chickpea wilt. The combination of *P. fluorescens* with the safer fungicides also reduces a wilt complex in the pigeon pea [129].

#### 4.2.3 Seed priming

Priming the seeds with biocontrol agents is an auspicious method to shelter seeds against numerous seedborne and soilborne pathogens. This is a technique responsible for inciting variations in the characteristics of plants while at the same time assisting in identical seed germination [130]. The PGPR also improve seed germination and sapling establishment [131], also recorded a ten-fold increase in antagonist inhabitant burden on the seeds that results in seed bio-priming by using antagonist bacteria, thus protecting the crop rhizosphere from many phytopathogen invasions. Field pea seed priming with *Pseudomonas aeruginosa, P. fluorescens,* and *B. subtilis* leads to a 20 % decrease in *Uromyces fabae* incidence under open field conditions [132]. Raguchander et al. [133] noticed that the seed pelting using *B. subtilis* efficiently managed a root rot caused by *Macrophomina phaseolina* in a soybean field.

#### 4.2.4 Foliar spray

Application of *Pseudomonas* spp. and *B. subtilis* on the leaves was recorded to decrease the number of rusts caused by *Uromyces phaseoli* in beans [134]. Similarly, foliar application and seed treatment of *P. fluorescens* (Pf1) also reduced the harshness of *Puccinia arachidis* under field conditions in groundnuts [135]. The capability of the entomopathogenic PGPR strains towards phylloplane colonization through a persistent mode was remarkable for the management of foliar pests in many crop plants [136]. Bacterial bioproducts were involved in root treatments, and they were applied via drenching or direct dipping [137]. Even bacterial biopesticides can be applied as foliar sprays, but this method of application greatly depends on the type of target organism, the infested crop, and the releasing system. Several *Bt*-based formulations were applied through foliar application to many crops. Ultra-low volume products of Bt successfully managed the insect pests of cotton and banana as well as successfully controlled the population of spruce bud worms [138].

### 4.3 Field efficacy of entomopathogenic bacteria

As mentioned earlier, entomopathogenic bacteria release several types of insect-killing proteins, which are sometimes pest-specific. In the year 1998, about 200 biopesticides produced from Bt were registered, among them, about 27 were effective against members of the Diptera and others were used for the management of insects belonging to the order Lepidoptera and Coleoptera [40]. Bt subsp. *kurstaki* by-products such as Javelin WG®, Biobit®, Deliver®, etc. were specific for the management of lepidopterans including *Plutella xylostella*, *Pieris rapae*), armyworm, *Ostrinia nubilalis* and *H. zea*. Bio-formulations of Bt subspecies aizawai (XenTari ® Agree® WG) were also reported to cause mortality in lepidopterans. Bt subsp. *tenebrionis* is found to be highly lethal against several leaf beetles, including *Leptinotarsa decimlineata*. Spinosad produced from *S. spinosa* is found to be deadly poisonous against a wide range of insects [[139], [140], [141]]. The byproducts emamectin benzoate and abamectin were formulated from the compound avermectins produced by *Streptomyces avermitilis*. These formulations show efficacy against a variety of insect nematodes and mites [142,143]. Bt var. *israelensis* and *B. sphaericus* were reported to have brilliant BCA against *Culex quinquefasciatus* and *Anopheles gambiae* larvae [144]. *Bacillus thuringiensis* var. *israelensis* was involved in the efficient management of *Aedes vexans, Aedes cataphylla Dyar,* and *Aedes communis* larvae [145]. Several insects and mites were excellently managed by *Burkholderia rinojensis*is [146]. The formulation of *Chromobacterium subtsugae* was also highly virulent against several insect orders, including Hemiptera, Diptera, Colepotera and Lepidoptera [58].

*Brevibacillus laterosporus* has been found to be effective against various insect orders, namely order Coleoptera, Diptera and Lepidoptera. Even they are found to be successful in the management of some plant pathogenic bacteria, fungi, molluscs, and nematode worms [147]. *Bdellovibrio bacteriovorus* has been applied to control the blackleg disease in potatoes caused by the bacteria *Erwinia carotovora* [148]. *Streptomyces aureus* was reported to manage the mite population. Ivermectin, a compound produced by *Streptomyces avermitilis,* is highly effective against nematodes and arthropods [149]. *Pseudomonas fluorescens* was reported to be effective against *Amrasca devastans* (leafhoppers) of *Bt* cotton [150]. Shrestha et al. [151] reported that Cry8Da toxin isolated from a noval *B. thuringiensis* subsp. *galleriae* was highly virulent against Alfalfa weevil (*Hypera postica*).

## 5 Biotechnological applications

Biotechnology is quickly expanding its share in biological sciences and also has a role in sustainable agriculture, which probably increases 70 % of agricultural production. People become aware of agricultural stress and food insecurity. They knew that sustainable agricultural practices were fundamental for future agricultural demand [152]. Despite the many challenges faced by the adoption of biopesticides, they are still useful without any negative impact. Bacterial biopesticides have potential benefits for agriculture and human health and do not have residue problem, which is an important issue for consumers. Biological control agents, such as EPB, are normally known for their lower-risk substances than chemical pesticides, and several benefits are related to their uses. Some bacterial biopesticides, such as *Pseudomonas*, *Bacillus*, *Xanthomonas, Rahnella,* and *Serratia* show different modes of action against the pathogens, such as hyperparasitism, competition, lysis, and predation [153]. Species of *Pseudomonas* and *Bacillus* have been used as biofertilizers that increase plant growth, yield, phosphorous, and zinc content in fruits and soils [154]. Several plant growth-promoting rhizobacteria (PGPR) include *Bacillus*, *Flaivobacterium*, *Azospirillum*, *Chromobacterium*, *Agrobacterium*, *Pseudomonas, Micrococcous*, *Azotobacter*, *Serratia*, *Caulobacter*, *Arthrobacter*, *Erwinia,* and *Burkholderia* [155], which are responsible for plant growth. These PGPR enhanced plant growth either directly by providing nutrients or indirectly by showing inhibitory effects on phytopathogens [156].

In the uninterrupted search to improve the efficiency of the identified biopesticides and to develop new biomolecules, recombinant DNA technology is being prescribed. Novel fusion proteins are now being developed next-generation biopesticides. This methodology only allows toxin (non-toxic against higher organisms) that is combined with the carrier protein and makes it only toxic to pests when consumed orally. It was also toxic when injected by the predator into the body of the target prey [157]. Fusion protein might be produced as the recombinant protein in the microbial system that may be scaled up in commercial formulations and industrial production. Several innovative approaches can be applied to develop biopesticides that are efficient, effective, and easily acceptable for pest control. One such fusion protein was developed by combining the toxic proteins of the tube-web spider (*Segestria florentina)* or Indian red scorpion (*Mesobuthus tumulus)* with *Galanthus nivalis* agglutinin (GNA), a snowdrop lectin that acts as a vehicle for the dispersal of toxin in insect midgut [158]. Delta endotoxin hybrids, such as fusion proteins as well as chimeric proteins, were formed with recent innovation in the biotechnological applications. Several chimeric toxic genes produced by such recombinant technology, including Cry1C, Cry1B, Cry1D, CryIA, and CryIG, are found to contribute to development and management of variety of insects. Production of transgenic plants is also possible only through these advanced biotechnological techniques.

### 5.1 Beneficial impact on agricultural and environmental sustainability

The rationale for the development and deployment of bacterial biopesticides for pest management is their environmental safety, specificity, and biodegradability. Some disease-causing agents, like bacteria and other microorganisms, are selected for their commercial development and have the ability to infect many species of insects. Therefore, the commercially available bacterial biopesticides are target-specific and have not been shown to infect other non-target organisms (Table 2). The biodegradable nature of these bacterial pesticides neither has any harmful effect on the environment nor enters the food chain. Without creating a negative impact on the environment, these bacterial biopesticides supply important tools that are extremely helpful for the control of diseases and pest populations. These are less toxic to bees, birds, and fish, as well as to wildlife, and help to maintain the population of beneficial insects that are rapidly declining in the environment [159].

**Table 2:** Commercial formulations of bacterial biopesticides available in the market.

Bacterial Biopesticides	Uses	Active Ingredient	Recommended doses	Target Organism
Bactur®WDG	Against foliage-feeding caterpillars	*Bacillus thuringiensis* var. *kurstaki*	0.5 g/lit	Caterpillars
Plant's buddy	It produces endotoxin (parasporal crystal) and exotoxin, which can make the pests stop feeding. The pests all die due to starvation, cell wall rupture and nerve poisoning.	*B. thuringiensis* var. *kurstaki*	10 ml/lit	Lepidopteran caterpillars
SM Srimalar Enterprises®	The active ingredient release enzymes that pierce the stomach lining of mosquito larvae. Also act as effective fungicide	*B. thuringiensis*	2–3 ml/lit	Mosquitoes, Black Flies and fungi
Bio larvicide	The crystal proteins produced by Bt are toxic to certain insect larvae	*B. thuringiensis* var. *kurstaki*	2–3ml/lit	Larvae
Utkarsh BT	Utkarsh BT acts by producing a protein that blocks the digestive system of the insect, effectively starving it; an infected insect will stop feeding within hours of ingestion and will die within days	*B. thuringiensis* var. *kurstaki*	2–3 ml/lit	Larvae
Mahastra	Effective in control of caterpillars	*B. thuringiensis* var. *kurstaki*	5-10 g/lit	Caterpillars
Thuricide®	Effective in control of caterpillars and worms	*B. thuringiensis* var. *kurstaki*	2 - 4 tsp. per gallon	Caterpillars and worms
Bactospeine®	Due to the toxic component, the insect intestines are affected and start decaying, leading to death within 24–72 h	*B. thuringiensis* var. *kurstaki*	1 g/lit	Larvae
Green Heal Larvicide	After entry into the host it paralyzes the digestive tract and cause death	*B. thuringiensis* var. *kurstaki*	5ml/lit	Caterpillars
VectoLex® Granules	A mosquito larvicide produces toxins inside the midgut and cause lysis of cells and larvae death	*B. thuringiensis sphaericus*	4 tsb/272 square feet	Mosquitoes
VectoMax® Granules	A mosquito larvicide produces toxins inside the midgut and cause lysis of cells and larvae death	*B. thuringiensis sphaericus*	30 lbs/ft3	Mosquitoes
VectoBac® WDG	A mosquito larvicide produces toxins inside the midgut and cause lysis of cells and larvae death	*B.thuringiensis* sub sp. *israelensis*	2–8 g/1000lit	Mosquitoes
Teknar® CG	A mosquito larvicide produces toxins inside the midgut and cause lysis of cells and larvae death	*B. thuringiensis* sub sp. *israelensis*	2.5–10.0lbs/acre	Mosquitoes
Gnatrol® SC	Bti protein crystals break down into toxins that bind to, and destroy, intestinal wall and larvae die quickly	*B. thuringiensis* sub sp. *israelensis*		Sciarid fly
Majestene®	Bionematicide affect RKNs life cycle in the plant and kill them	Burkholderia rinojensis	9.5 L/ha	Root-knot nematodes (RKNs)
Tracer™ 120 SC	Used primarily for the control of chewing and scratching insects	*Saccharopolyspora spinosa*	0.375 ml/lit	Insects
BioNemagon™	Infects and kills both larvae and adult stage of many plant pathogenic nematodes	*Bacillus firmus*	10–30 kg/acre	Nematodes
Grandevo ® WDG	Shows multiple modes of action: repellency, ingestion, and reproduction disruption.	*Chromobacterium subtsugae*	3 lbs/100 gallons	Thrips Whiteflies
Novodor@	Produces protein crystals that kills the larvae	*Bacillus thuringiensis* subsp*. tenebrionis*	3 to 4 quarts per acre	Colorado Potato Beetle and Elm Leaf Beetle,
Agree-WP	Strong and effective stomach action leads to death of the insect	*B. thuringiensis* subsp*. aizawai*	0.5–2.0lbs/acre	Loopers, Armyworm

Biopesticides may contain microbial antagonists but not synthetic chemicals that might have a positive impact on the environment [160]. These microbial antagonists are used to manage the populations of the different plant pathogens. The *Pseudomonas* genus was studied widely and tested along with the microbial antagonists [161]. On the other hand, the outcomes of Bt have been well acknowledged for the environment. Spores of Bt are released into the soil from the decomposing dead insects after they have been killed in to the soil and inactivated at a pH below 5.1. Microbial pesticides such as *Bt* are classified as immobile because they do not move or leach with the groundwater because of their fast breakdown and low toxicity, which do not have any bad impact on aquatic ecosystem. A shortage of receptors that bind to *Bt* toxins in mammals, causes the degradation of *Bt* toxins in the human digestive system and makes them harmless for human beings. The security of the *Bt* toxins in terms of toxicity and allergenicity towards mammals and other non-target organisms is well accepted. *Bt*-sprays are harmless to non-target organisms and soil microorganisms like protozoa and fungi, and other organisms such as crustacea, mollusca, arachnida, salamanders, collembola, aquatic insects, honeybees, parasitoids, earthworms, predators, birds, and mammals. Frequently, studies have discovered that biocontrol Pseudomonads are broadly distributed in agricultural soils. Their multiple crop habitats and different soil factors can affect their abundance and activities [162]. The bacterial biopesticides are useful for managing the population of insect pests and plant pathogens as a result of their beneficial effects. Thus, the application of biopesticides supports stability and sustainability in agroecosystem because they are eco-friendly by nature.

## 6 Limitations and challenges

It is very well known that India has an agriculture-based economy, and 70 % of the Indian population relies on agriculture for its food. Due to the emergence of the green revolution in the 1970s, use of chemical pesticides has increased tremendously. But now people are much more aware of the health hazards caused by these chemical pesticides. So efforts have been made to produce some alternatives that should be economic and eco-friendly. The use of bacterial biopesticides is the best option in place of chemical synthetic pesticides.

Although the application of these biopesticides results in better production and plays an important role in sustainable agriculture. However, the use of these biopesticides sometimes becomes challenging. Several limitations have been faced by the biopesticides industry in India. These limitations include, it is necessary to verify the microorganisms (from which the biopesticides developed) for biosafety; the shelf life of the biopesticides and their effectiveness are quite slow; sometimes *in vitro* culturing might be successful, but in the open field, the experiment may fail; as bacterial biopesticides involve living microorganisms, so it is quite challenging to maintain the efficacy of the biopesticides in the field as there must be fluctuations in the abiotic factors of temperature, pH, humidity, sunlight exposure, rain, wind; a large proportion of biopesticides have been required to spray even a smaller area; the registration process for formulation is also quite a challenging task, as data about the toxicity, chemistry and packaging of the product is required; and as the farmers are well aware of the doses and uses of chemical pesticides, they don't want to waste time on biopesticides. Moreover, the doses of the biopesticides are not definite.

## 7 Conclusions

Agriculture is badly affected by various insect pests that leads to poor quality produce and yield reduction. Since the 1960s, collective methods to manage the population of various pests have been the adopted, including chemical and synthetic pesticides. These synthetic pesticides were accepted in the 1940s with the discovery of dichlorodiphenyltrichloroethane (DDT), followed by the other organophosphates and the carbamate pesticides. After that, Green Revolution Technology increased food production mainly in developing countries through the intensive use of pesticides and chemical fertilizers. Although, the usage of these chemicals caused detrimental effects on water quality, produce quality, soil health and established many problems such as genetic variation, insect resistance, presence of toxic residues on food and feed etc. Knowing the inimical effects of these agrochemicals, like outbreaks of secondary pests, pest resurgence, pesticide resistance, and pesticide residues in produce, water, soil, and air, it is now necessary to develop substitutes for these synthetic chemical agro-inputs. The necessity of the day is to produce the maximum from diminishing natural means or resources and to protect agricultural produce, mainly post-harvest losses without disturbing the natural environment. The use of biopesticides is eco-friendly, easily biodegradable, has no effect on the environment, and plays a major part in dealing with the challenges in agriculture in a sustainable manner. Biopesticides are gaining attention due to the advantages associated with them, as they are target-specific and hence do not affect beneficial organisms such as natural enemies. They are also helpful in small quantities, and their uses endorse sustainable pest management, thus contributing towards sustainable agriculture. Biopesticides are promising alternatives for the management of insect pests and other environmental problems.

## CRediT authorship contribution statement

**Preety Tomar:** Writing – original draft. **Neelam Thakur:** Writing – review & editing. **Samiksha Jhamta:** Writing – original draft. **Sohini Chowdhury:** Writing – review & editing. **Monit Kapoor:** Writing – review & editing. **Sangram Singh:** Writing – review & editing. **Sheikh Shreaz:** Writing – review & editing. **Sarvesh Rustagi:** Writing – review & editing, Conceptualization. **Pankaj Kumar Rai:** Writing – review & editing. **Ashutosh Kumar Rai:** Writing – review & editing. **Ajar Nath Yadav:** Writing – review & editing, Supervision, Conceptualization.

## Declaration of competing interest

The authors declare that they have no known competing financial interests or personal relationships that could have appeared to influence the work reported in this paper.
